# CLINICAL AND FUNCTIONAL EVALUATION OF WRISTS AND HANDS OF SPINAL CORD INJURED PATIENTS

**DOI:** 10.1590/1413-785220243201e264175

**Published:** 2024-03-22

**Authors:** Cíntia Kelly Bittar, Isabela Ferreira Perucci, Danillo Nagel Signorini, Mariana Buratti Mascarenhas, Orcizo Francisco Silvestre, Alberto Cliquet

**Affiliations:** 1 Pontifícia Universidade Católica de Campinas, School of Medical Sciences, Campinas, SP, Brazil.; 2 Universidade Estadual de Campinas, Department of Orthopedics, Rheumatology and Traumatology, Campinas, SP, Brazil.

**Keywords:** Osteoarthritis, Pain, Muscle Strength, Osteoartrite, Dor, Força Muscular

## Abstract

**Introduction:**

The inability of the spinal cord to propagate sensory and motor stimuli as a result of the disruption of the nerve tracts is called spinal cord injury.

**Objective:**

This study analyzes clinically and radiologically the hands and wrists of spinal cord injured patients, evaluating their motor and sensitive functionality, in order to determine if these patients are more likely to develop degenerative alterations.

**Methods:**

14 patients (8 paraplegics and 6 tetraplegics) were evaluated, undergoing anamnesis and clinical examination - a scale of muscular strength (MRC - Medical Research Council) and the amplitude measurement of the movement with a manual goniometer (ROM), were used for objective evaluation - and x-ray exams. The results were compared with pre-existing data from other studies.

**Results:**

When asked, only one of the 14 observed patients complained about constant wrist pain, described as level 3 (weak to moderate), based on the visual analog scale (VAS). The motor evaluation, MRC and ROM divided the group of patients into two subgroups: paraplegic and tetraplegic patients. The x-ray analysis showed, based on Kellgren and Lawrence classification, that all exam images fit grades 1 or 2 of osteoarthritis and osteoarthrosis.

**Conclusion:**

In conclusion, spinal cord injured patients showed none or minimal clinical and radiological signs of osteoarthritis on hands or wrists. Overall, the hands and wrists of spinal cord-injured patients behave similarly to noninjured patients. ***Level of Evidence III; Retrospective Comparative Study***.

## RESUMO

**Introdução:**

A incapacidade da medula espinhal de propagar estímulos sensoriais e motores como resultado do rompimento das vias nervosas é chamada de lesão da medula espinhal.

**Objetivo:**

Este estudo analisa clínica e radiograficamente mão e punhos de pacientes lesados medulares, avaliando função motora e sensitiva, a fim de determinar se estes pacientes estariam mais propensos a alterações degenerativas.

**Métodos:**

14 pacientes (8 paraplégicos e 6 tetraplégicos) foram avaliados, passando por anamnese e exame clínico - sendo escala de força muscular (MRC - Medical Research Council) e a medição da amplitude de movimento com um goniômetro manual (ROM) foram utilizados para análise objetiva - e radiografias. Os dados obtidos foram comparados com literatura preexistente.

**Resultados:**

Quando questionados, apenas um dos 14 pacientes observados referiu dor crônica nos punhos, descrita como nível 3 (fraca a moderada), baseada na escala visual analógica. A avaliação motora, MRC e ROM dividiram os pacientes em 2 subgrupos: pacientes paraplégicos e tetraplégicos. A análise radiográfica mostrou, baseada na classificação de Kellgren e Lawrence, que todas as imagens se encaixam nos graus 1 ou 2 de osteoartrite e osteoartrose.

**Conclusão:**

Conclui-se, então, que pacientes lesados medulares apresentam nenhuma ou mínimas alterações clínicas e radiológicas para osteoartrite ou osteoartrose de punhos ou mãos. Ou seja, no geral, mãos e punhos de pacientes lesados medulares comportam-se como os pacientes sem lesão medular. ***Nível de Evidência III; Estudo Retrospectivo Comparativo.***

## INTRODUCTION

The inability of the spinal cord to propagate sensory and motor stimuli as a result of the disruption of the nerve tracts is called spinal cord injury. Motor and sensory dysfunctions are observed in the body segment below the level of the injury, affecting the locomotor system, viscera and homeostasis of the body, by deregulating the sympathetic nervous system.^1,2^

They can be complete or incomplete, which implies the clinical picture of the spinal cord injury, because in partial injury, muscle groups and sensory areas may be preserved, while in total injury there is total loss of these functions, and the entire body segment below the injury is affected.^1^

Spinal cord injury can be: tetraplegia, which consists of the loss of motor and/or sensory function affecting both the lower and upper limbs, trunk and pelvic organs, and paraplegia, which is the loss of function of the thoracic, lumbar and sacral segments, including the lower limbs.^2^

The most frequent traumatic causes are gunshot wounds, automobile accidents, falls from heights, and diving in shallow water. Non-traumatic lesions are tumors, infections, vascular changes, congenital malformations, or degenerative or compressive processes. In Brazil, traumatic spinal cord injuries are unknown, as they are not reported, and it is estimated that the annual incidence is approximately 40 cases/million in inhabitants.^Isaiah 1:3-5^

Spinal cord injury represents an important disabling syndrome since the sequelae become highly limiting in the physical and socioeconomic spheres. Therefore, rehabilitation aims to reintegrate him into the activities of society.^6-8 years^

Orthopedic comorbidities are common and mainly affect the joints of the upper limbs, especially paraplegic ones. They are considered secondary to the increase in stress caused using these upper limbs, as they are recruited to try to compensate for the lack of lower limbs; Paraplegics use the strength of the upper limbs to move, either for body transfer and accommodation or for propulsion in a wheelchair and walker. The upper limbs of patients with spinal cord injury are recruited with greater frequency and strength when compared to patients without spinal cord injury, which results in greater stress and overload on these limbs. Studies indicate that the most important pain complaints are from the shoulder, elbow, wrist and hand.^9-12^

This study highlights these comorbidities related to the wrist and hand joints in paraplegic and quadriplegic patients and compares clinical and radiological criteria with normality parameters in the literature.

## METHODS

The study was conducted at the spinal cord injury outpatient clinic of the University Hospital, SP, Brazil, in 2020/2021 and approved by the CAE ethics committee: 23257613.4.0000.5404.

A total of 14 patients were evaluated, eight paraplegic and six quadriplegics, aged between 28 and 68 years (mean 45 years). Of the patients, 12 were male and two were female. The ASIA test was performed to classify spinal cord injuries. The ASIA of each patient was related to the respective mechanism that led to the spinal cord injury. First, a subjective evaluation was performed, which consisted of a detailed anamnesis to evaluate complaints associated with pain, and an inspection of the patients’ hands, looking for deformities, calluses, and retractions.^13.14^

Then, the sensory and motor evaluation of the patients’ hands and wrists was performed bilaterally to determine the preservation of certain functions of the median, radial and ulnar nerves. The patients were separated into two subgroups, differentiating paraplegics (8 individuals) and quadriplegics (6 individuals).

The physical-motor examination seeks to identify lesions in the median nerve, which result in paralysis or paresis of the muscles responsible for hand abduction and the ability of the thumb to oppose or makes it impossible or difficult to grasp between the thumb and the other fingers of the hand. Injuries to the radial nerve, which cause difficulty grasping objects as they affect the extension of wrists and fingers. On the other hand, ulnar nerve injuries result in claw deformity in the little and ring fingers (hyperextension of the metacarpophalangeal joints and flexion of the interphalangeal joints).^15th^

To analyze the preservation of the sensory functionality of the superior peripheral nerves, we took into account the area of cutaneous innervation of these nerves, where the radial nerve is responsible for the cutaneous innervation of the lateral region of the dorsum of the hand, dorsum of the thumb, proximal phalanges of the index and middle fingers, and a large part of the posterior region of the arm and forearm; cutaneous innervation of the lateral half of the palm, palmar surface of fingers l-III and half of the IV finger, and dorsal surface of the middle and distal phalanges of the Il and Ill fingers and lateral half of the IV finger; and, finally, the ulnar nerve, responsible for the cutaneous innervation of the hypothenar eminence and the medial region of the dorsum of the hand.^15th^

The superficial flexor, deep flexor, superficial extensor and deep extensor muscles of the hand were analyzed using the MRC-Medical Research Council as a parameter.^16^

During the physical examination, the range of motion (ROM) was measured using a goniometer to assess the angles of supination/radioulnar pronation and flexion/extension and abduction/adduction of the wrist. The values considered normal for joint range of motion are:^17^

Supination and radioulnar pronation: 0°-90°

- Wrist flexion: 0°-90°
- Pulse extension: 0°-70
- Pulse abduction: 0°-20°
- Wrist adduction: 0°-45

**Figure 1:**
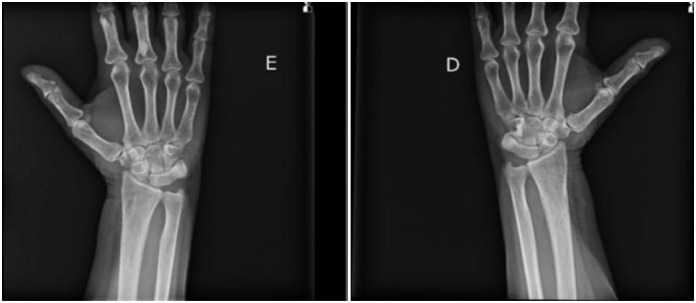
Anteroposterior view: radiographic image of the left and right hand and wrist, respectively.

**Figure 2:**
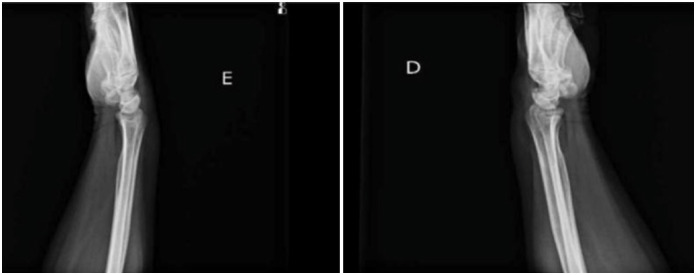
Lateral view: radiographic image of the left and right hand and wrist, respectively.

Angular and mobility measurements were performed by manual methods by three examiners. Each examiner performed three measurements and the mean was. (Figures 1 and 2) considered in relation to the 9 measurements performed, this one was repeated for each angulation given by the manual goniometer. This method was performed due to the lack of a digital goniometer.

The study used radiographic examinations of the wrist in the anteroposterior and lateral positions for analysis and classification of the stages of osteoarthritis to compose the radiographic analysis of the patients. As exemplified below (both images are of the same patient):

The Kellgren and Lawrence system was used for the classification, which, according to the appearance of osteophytes in the marginal joints, periarticular ossicles or pseudocysts and joint reduction associated with subchondral bone sclerosis and marginal bone alteration, categorizes the radiological image and, consequently, the osteoarthritis of the patients. According to Kellgren and Lawrence’s classification, OA can be divided into the following categories:^18^

0: no change, no osteoarthritis
1: some alteration may be found, indicating the onset of osteoarthritis, but without clinical repercussions
2: minimal and confirmed diagnosis of osteoarthritis, but minimal severity
3: Moderate
4: Record

All analyses were performed bilaterally.

For the statistics, the data obtained according to these methods, their maximum values, minimum values, standard deviation and mean were considered.

## RESULTS

Fourteen patients with a average age of 45 years (ranging from 28 to 68 years) participated in the study; 2 were female and 12 were male. Level and characteristic, according to the ASIA standard categorization, as well as the mechanism of this lesion, are described in the table below. (Tabela 1)^14^

**Table 1:** The spinal cord injury is classified according to the international standardization determined by the American Spinal Injury Association-ASIA.

ASIA	injury mechanism
C3A	fall from height
C4A	car accident
C4B	car accident
C5A	shallow water diving
C6B	gunshot wound
C6B	shallow water diving
T4A	car accident
T4B	gunshot wound
T7A	fall from height
T7A	fall from height
T7A	car accident
T8A	car accident
T8D	spinal cord infarction
T12A	car accident

ASIA "A") Complete Injury: Without preservation of motor and sensory functions in the sacral segment S4-S5.

ASIA "B") Incomplete Injury: Loss of motor function, but preserved sensory function below the neurological level and includes sensitivity of sacral segment S4-S5.

ASIA "D") Incomplete Injury: Preserved motor function below neurological level, and more than half of the key muscles below neurological level have a strength grade of 3 or more.^14^

During the anamnesis, one patient interviewed reported constant wrist pain, which he described as a level 3 pain (weak to moderate) according to the Visual Analog Pain Scale. The other 13 study participants reported no complaints of significant severity and constancy regarding pain. Also, no relevant deformities such as calluses and retractions were observed.^13^

Then, the physical examination was performed to evaluate the preservation of peripheral nerves of the upper limbs, following the distinction made between paraplegic and paraplegic patients. Among the paraplegic patients, only one of the eight patients observed presented some functional limitation, and a functional dysfunction was described only in the median nerve. For the group of quadriplegic patients, it was not possible to identify any level of functionality and preservation of these nerves, not having any type of movement or sensibility.

About the Muscle Strength Evaluation Scale (MRC-Medical Research Council), it is important to separate the patients into groups. For the paraplegic patients, the following averages were found. (Tabela 2)

**Table 2:** MRC-Medical Research Council (0) Absence of contraction; 1) Outline of muscle contraction; 2) Active movement, but not overcoming gravity; 3) Active movement against gravity; 4) Active movement against gravity and with resistance; 5) Complete mobility, overcoming additional resistance.^16^

Muscles	AveragesMRC	Maximum value Mrc	Minumum value Mrc	Standard deviation
Left flexor superficialis	4,8	5	4	0,44
Right flexor superficialis	5	5	5	0
Left flexor profundus	4,8	5	4	0,44
Right flexor profundus	5	5	5	0
Left extensor superficialis	4,8	5	4	0,44
Right extensor superficialis	5	5	5	0
Left extensor profundus	4,4	5	3	0,89
Right extensor profundus	4,4	5	3	0,89

In contrast, as already observed in the previous analysis, those quadriplegic patients presented mean, and standard deviation, 0 (zero) in the muscle strength evaluation, showing no evidence of muscle contraction - an expected characteristic due to the high spinal cord injury.

Then, the measurement of range of motion (ROM) was analyzed, with the goniometer as an instrument. Again, since this is an analysis of the active hand positioning ability, the data obtained for the quadriplegic patients were 0 (zero), mean and standard deviation. For the paraplegic patients, the results of the averages obtained were as follows. (Tabela 3)

**Table 3:** The normal values for joint range of motion are: radio-ulnar supination and pronation: 0°-90°; wrist flexion: 0°-90°; wrist extension: 0°-70°; wrist abduction: 0°-20°; wrist adduction: 0°-45°.^17^

Measure	Angulation Average	Standard Deviation
Left radioulnar supination	88,4°	1,81
Right radioulnar supination	87,2°	4,08
Left radioulnar pronation	86,8°	4,43
Right radioulnar pronation	86°	4,18
Left wrist flexion	75,2°	11.54
Right wrist flexion	77°	13.85
Left wrist extension	62,6°	11,58
Right wrist extension	56,8°	5,89
Left wrist abduction	33,8°	15,56
Right wrist abduction	34,6°	12,05
Left wrist adduction	56,2°	10,75
Right wrist adduction	55,2°	9,80

Finally, radiographic examination was performed to investigate OA in these patients, which indicated the following results described below. (Tabela 4)

**Table 4:** Kellgren-Lawrence Arthrosis Rating Scale - 0) None; 1) Questionable 2)Minimal; 3)Moderate; 4)Severe.^18^

Patient asia	Years of age	Osteoartrite xr left	Osteoartrite xr right
T8A	36	1	1
T7A	68	1	1
T12A	28	1	1
T8D	61	1	1
T4B	29	1	1
C4B	58	2	1
C4A	50	2	2
C3A	42	2	2
T7A	42	2	2
C5A	35	2	1
C6B	35	1	1
T7A	43	1	1
T4A	55	1	2
C6B	52	1	1

Among the patients analyzed, radiographs showed that from the total of 16 wrists and hands belonging to the paraplegic group, 81.25% fit OA stage 1 and 18.75% stage 2. For the quadriplegic group, the analysis showed a sample of 50% with OA stage 1, and the rest of them were classified as OA stage 2. A total of 12 wrists and hands of quadriplegic patients were counted.

## DISCUSSION

The objective of this study was to carry out, through the methodologies presented, a functional and motor evaluation of patients with spinal cord injury and to try to compare whether their daily activities, with the use of wheelchairs and repetitive movements, favored the development of osteoarthritis; To evaluate the relevance of the data obtained to confirm whether, in fact, the use of wheelchairs in patients with spinal cord injury favored the development of joint problems in the wrists and hands.

Most of the patients were male, young adults, with equal distribution in relation to the height of the lesions and different mechanisms for these lesions.

With the results obtained, it was possible to perceive that patients with spinal cord injury have mild or absent clinical and radiographic alterations in the wrists and hands. Among the patients analyzed, only 32.14% had signs of osteoarthritis (category 2), and the remainder were classified as category O or 1 osteoarthritis, which presented mild alterations, without a definitive diagnosis and without clinical repercussions worthy of note.

In the functional and motor evaluations carried out during the development of the research, the following will be discussed:

In the anamnesis, only one of the patients reported complaints associated with pain, such as inflammatory pain, or worsening at rest and with some morning stiffness, which improved after 30 minutes of movement. The remaining patients did not complain of significant pain.

In the motor physical examination, the objective was to identify lesions of the median, radial and ulnar nerves, the results found corroborated findings in the literature. The clinical findings were consistent with those expected for the ASIA examination, which is the characteristic and height of the spinal cord injury, as described by Mazurek, Michael T.MD et al (2001) and Maynard FM Jr et al (1997).^14.15^

According to the authors, during the physical examination, the preservation of the sensory function of the superior peripheral nerves was analyzed, as well as in the motor evaluation, the sensory results obtained did not diverge from those expected, as described by Mazurek, Michael T.MD et al (2001) and Maynard FM Jr et al (1997). The clinical findings were consistent with those expected for the disease, as indicated by the existing literature, i.e., the characteristic and height of the spinal cord injury.^14.15^

James, M. A. (2007) postulated in relation to the "Muscle Strength Rating Scale (MRC-Medical Research Council)", important for the diagnosis of weakness the study showed that the highest scale observed was 5, corresponding to the normal parameters of force against the gravitational force and the applied resistance, and the smallest scale was 3, which demonstrates active movement against the force of gravity, but not overcoming the added resistance. In patients with a scale below 5, the results were consistent with the ASIA, i.e., the characteristic and height of the spinal cord injury, and there was no correlation between the change in the behavior of the functional status and muscle strength with the objective of the research. In the quadriplegics, the mean and standard deviation were 0 (zero) in the evaluation of muscle strength, with no evidence of muscle contraction - a characteristic expected due to the high spinal cord injury.^16th^

Wrist goniometry, an evaluation technique used to determine the range of motion restrictions of these patients. As previously noted, the analysis of all patients is within normal range of motion values. We did not observe any representative alteration in the reduction of joint mobility in the patients studied, with the exception of the quadriplegic group, which, as it was an analysis of the ability to actively position the active hand, the data obtained for the quadriplegic were 0 (zero), mean and standard deviation. Therefore, during the evaluation, the results on range of motion also did not diverge from the expected results, according to data found by Marques, Amélia Pasqual (2003) and Maynard FM Jr et al (1997). The clinical findings were consistent with those expected for ASIA, i.e., the characteristic and height of the spinal cord injury.^14.17^

Radiographic analysis of these patients was performed in order to look for any sign indicative of osteoarthritis and to use it as a comparison with the clinical data previously obtained. The classification of wrist osteoarthritis, therefore, indicated that, of the 28 radiographs analyzed, 67.86% of the cases fell into categories 0 and 1, i.e., no radiographic alterations or, at most, minimal alterations, without a closed diagnosis of osteoarthritis or any clinical repercussion. The remaining 32.14% fall into stage 2, i.e., with evidence of osteoarthritis, but of low grade.^18th^

It is noteworthy that most of these patients are extremely debilitated, one of the possible causes of osteoarthritis can facilitate the development of preventive strategies, seeking to improve the quality of life of these individuals. Delaying or preventing the onset of osteoarthritis requires lifestyle changes that can minimize more serious clinical problems, especially in patients with spinal cord injury, in whom the approach is more complex, since therapeutic resources are scarcer and not always accessible.^19.20 am^

The importance of this study lies in the use of this material as an object of analysis for other research related to the theme, thus serving as a comparative support for the composition of other research in the area.

Some limitations were: angular and mobility measurements were performed by the manual method, which was used due to the lack of a digital goniometer. No study on the hand and wrist pattern in spinal cord injury was found in the literature. This paucity of publications suggests that more studies are needed to define standards and compare different treatments for this group of patients.

## CONCLUSION

We conclude that the use of wheelchairs and repetitive movements in patients with spinal cord injury does not favor the development of high degrees of osteoarthritis. The evaluations and tests applied to 14 patients with spinal cord injury show that the results followed the normal standard, with the exception of the quadriplegic group, in which the evaluations could not be performed due to the inability to perform and measure range of motion, muscle strength and nerve preservation.

The rest of the patients did not present significant alterations that express a relationship between the use of wheelchairs and the development of osteoarthritis. Therefore, the wrists and hands of these patients behave similarly to uninjured individuals.
